# Evaluating Protein Extraction Techniques for Elucidating Proteomic Changes in Yeast Deletion Strains

**DOI:** 10.3390/proteomes13030028

**Published:** 2025-07-01

**Authors:** Valentina Rossio, Joao A. Paulo

**Affiliations:** Department of Cell Biology, Harvard Medical School, Boston, MA 02115, USA

**Keywords:** yeast proteomics, gene deletion, protein extraction, SUMOylation, mass spectrometry, bead-based lysis

## Abstract

Background: Alterations in protein abundance profiles in yeast deletion strains are frequently utilized to gain insights into cellular functions and regulatory networks, most of which are conserved in higher eukaryotes. Methods: This study investigates the impact of protein extraction methodologies on the whole proteome analysis of *S. cerevisiae*, comparing detergent-based lysis versus mechanical lysis with silica beads. We evaluated the proteomic profiles of wild-type and two yeast deletion strains, *siz1*Δ and *nfi1*Δ (*siz2*Δ), which are SUMO E3 ligases. Combining isobaric TMTpro-labeling with mass spectrometry using real-time search MS3, we profiled over 4700 proteins, covering approximately 80% of the yeast proteome. Results: Hierarchical clustering and principal component analyses revealed that the choice of protein extraction method significantly influenced the proteomic data, overshadowing the genetic variances among these strains. Notably, the detergent-based lysis showed superior performance in extracting proteins compared to mechanical lysis. Despite minimal proteomic alterations among strains, we observed consistent changes regardless of the lysis strategy in proteins such as Ino1, Rep1, Rep2, Snz1, and Fdh1 in both SUMO E3 ligase deletion strains, implying potential redundant mechanisms of control for these proteins. Conclusion: These findings underscore the importance of method selection at each step of sample preparation in proteomic studies and enhance our comprehension of cellular adaptations to genetic perturbations.

## 1. Introduction

Changes in protein abundance profiles in yeast deletion strains are often used to understand and elucidate cellular functions and regulatory networks. The yeast *S. cerevisiae*, a well-established single-cell eukaryotic model organism, provides a framework for such studies due to its conservation of cellular pathways with higher eukaryotes and its inherent genetic flexibility [1]. The use of deletion strains, particularly, allows for the detailed investigation of gene function and its impact on the global proteome [2]. Proteomic analysis in yeast relies heavily on the method used for protein extraction, a crucial step that influences downstream protein detection and quantification. The importance of method selection for protein extraction/lysis in proteomics is underscored by studies showing that extraction efficiency and protein yield significantly affect downstream analytical outcomes, including mass spectrometry-based quantification [3,4,5,6]. Traditional methods of mechanical lysis, such as bead-based lysis that can disrupt strong cell walls, may have barriers to their use, including the availability of specialized equipment [7]. Bead-based lysis has shown promise in yielding a broad spectrum of proteins, thus providing a comprehensive view of the proteome. This method, however, requires careful optimization to balance protein integrity with extraction efficiency. Furthermore, the harshness of mechanical lysis can impact weak protein interactions, while the heat generated by mechanical force can affect labile post-translational modifications, which are essential for certain applications. Recent advancements highlight the potential of detergent-based lysis that offers simplicity, convenience, and may more comprehensively capture proteins, especially those that are membrane-bound [8].

In yeast proteomics, detergent-based lysis methods are widely employed to disrupt cell walls and solubilize proteins. These detergents are classified into ionic, non-ionic, and zwitterionic types. Ionic detergents, such as sodium dodecyl sulfate (SDS), excel at solubilizing membrane-bound proteins due to their strong denaturing properties but may disrupt protein structures, limiting their use in functional studies [9]. Non-ionic detergents, like Triton X-100, are gentler, preserving protein interactions and activity, though they are less effective for extracting hydrophobic proteins [10]. Zwitterionic detergents, such as CHAPS, offer a balance by maintaining protein solubility without disrupting native conformations, but they can be cost-prohibitive and less efficient for highly hydrophobic proteins [11].

Here, we compare side by side bead-based lysis and a detergent-based extraction (the Y-PER reagent [12]) in their ability to elucidate the proteomic profiles of wild-type and two *S. cerevisiae* deletion strains, *siz1*Δ and *nfi1*Δ. Siz1 (SAP and mIZ-finger domain 1) and Nfi1 (Neck Filament Interacting 1) or Siz2 are two paralog SUMO (Small Ubiquitin-like Modifier) E3 ligases sharing ~27% sequence identity and ~35% homology. They play significant roles in SUMOylation, which attaches the small molecule SUMO to target proteins, affecting their function, localization, and stability [13,14,15]. Specifically, Siz1 is considered the main SUMO E3 ligase in *S. cerevisiae*. By evaluating the global protein abundance profiles of these two gene deletion strains with wild-type, we aim to understand better the cellular pathways influenced by each SUMO ligase, their associated proteome remodeling, and if they act specifically or redundantly. In fact, the impact on the proteome of these two E3 SUMO ligases has not been yet directly compared. Our approach integrates TMTpro-labeling with advanced mass spectrometry using the Orbitrap Eclipse mass spectrometer and real-time search MS3 (RTS-MS3) to ensure precise and accurate relative quantification across all samples. Through this comparison, we aim to establish an optimized workflow for studying proteomic changes in genetically modified yeast strains. Our dataset will also help in characterizing the function of SUMOylation in controlling protein stability and cellular functions in budding yeast. Ultimately, our findings will enhance the understanding of methodological impacts of lysis techniques on proteomics and provide a framework for the analysis thereof.

## 2. Materials and Methods

### 2.1. Materials and Yeast Strains

ThermoFisher Scientific (Rockford, IL, USA) provided the protease inhibitors, trypsin, tandem mass tag isobaric labeling reagents, Y-PER yeast protein extraction reagent, and BCA kit. Lys-C protease was from Fujifilm Wako (Richmond, VA, USA). Mass spectrometry-grade organic solvents and water were purchased from J.T. Baker (Center Valley, PA, USA). StageTip Empore-C18 disks were supplied from CDSanalytical (Oxford, PA, USA), while Sep-Pak cartridges (50 mg) were obtained from Waters (Milford, MA, USA). The *S. cerevisiae* strains analyzed in this study (wild-type, *siz1*Δ, and *nfi1*Δ) were isogenic to BY4742 genetic background (*his3*Δ*1*, *leu2*Δ*0*, *lys2*Δ*0*, *ura3*Δ*0*) and were obtained from Horizon Scientific (Cambridge, UK). These yeast strains were grown in YPD media (1% yeast extract, 2% bactopeptone, and 2% glucose) sourced from SunriseScientific (Knoxville, TN, USA).

### 2.2. Growth Conditions

*S. cerevisiae* cultures were initially grown at 30 °C overnight in YPD medium. On the following day, the cultures were split into triplicates and diluted with fresh YPD medium (OD_600_ = 0.15). After approximately 5 h of incubation, cells were collected by centrifugation at 2000 g for 2 min. The resulting pellets were washed with water, and during the final wash step, the samples were divided in two for the two different lysis protocols. Following aspiration, the cell pellets were frozen in liquid nitrogen and stored at −80 °C until subsequent proteome analysis by mass spectrometry.

### 2.3. Cell Lysis and Protein Extraction

Yeast cell lysis was performed using either the commercial Y-PER buffer or a conventional mechanical method (bead beating). For the reagent lysis, cells were lysed by adding the Y-PER yeast protein extraction reagent following the manufacturer’s instructions. Alternatively, mechanical lysis was performed in a cold room using bead beating for five 30 s cycles with intermittent rest periods. After lysis, DNA was sheared by passing the lysates through a 21-gauge needle twelve times to facilitate the release of proteins bound to chromatin. Protein concentrations were quantified using a BCA assay, adhering to the manufacturer’s protocol. Reduction of proteins was achieved with 5 mM tris(2-carboxyethyl)phosphine (TCEP) for 20 min, followed by alkylation with 10 mM iodoacetamide for 20 min in absence of light and finally quenched with 10 mM dithiothreitol (DTT) for 20 min, always in absence of light. Chloroform–methanol precipitation on 100 µg of protein from each sample was performed, as described previously [16,17].

### 2.4. Protein Digestion, TMT Labeling, and Sample Processing

Protein samples were first digested with Lys-C overnight at 24 °C, followed by a second digestion with trypsin for 6 h at 37 °C. Each digestion used 1 µg of enzyme per 100 µg of protein. After digestion, acetonitrile was added to reach a final concentration of 30%, and the appropriate TMTpro labeling reagent was added to each sample for one hour. Before continuing sample processing, labeling efficiency was verified as described previously [18,19]. Hydroxylamine (final concentration ~0.3%) was added to each sample to terminate the labeling reaction, and the samples were incubated again at room temperature (15 min). Finally, labeled samples were combined in equal proportion and desalted using a 50 mg Sep-Pak solid-phase extraction column [20].

### 2.5. Basic pH Reversed-Phase (BPRP) Fractionation—Peptide Pre-Fractionation Was Carried out Using Basic pH Reversed-Phase (BPRP) High-Performance Liquid Chromatography (HPLC)

An Agilent 1260 system (Lexington, MA, USA) with an Agilent 300 Extend C18 column (3.5 μm particles, 250 mm length, and 2.1 mm ID) was employed. Peptides were separated over a 60 min linear gradient from 5% to 35% acetonitrile in 10 mM ammonium bicarbonate (pH 8) at a flow rate of 0.25 mL/min. Ninety-six fractions were collected and pooled into twenty-four superfractions. For further analysis, these were acidified to 1% formic acid, desalted using StageTips, dried by vacuum centrifugation, and reconstituted in 5% acetonitrile with 5% formic acid.

### 2.6. Liquid Chromatography and Mass Spectrometry Analysis

Mass spectrometry data were acquired on an Orbitrap Eclipse mass spectrometer coupled to a Neo Vanquish liquid chromatograph (both from Thermo Fisher Scientific, San Jose, CA, USA). Peptides were separated on a 100 µm inner diameter microcapillary column packed with ~35 cm of Accucore C18 resin (2.6 μm, 150 Å, Thermo Fisher Scientific). Approximately 1 μg of peptides was loaded per analysis. Separation occurred over a 90 min gradient of 5–29% acetonitrile in 0.125% formic acid at a flow rate of 400 nL/min. The acquisition began with an MS1 scan (Orbitrap; resolution 60,000; mass range 350–1350 *m*/*z*; AGC target 100%; auto maximum injection time). MS2 spectra were obtained via collision-induced dissociation (CID) in the quadrupole ion trap (turbo scan rate; AGC 2E4; NCE 35; maximum injection time 35 ms). MS3 analysis used high-energy collision-induced dissociation (HCD) with Orbitrap detection (NCE 55; AGC 200%; maximum injection time 200 ms; resolution 30,000; TurboTMT enabled). Real-time search (RTS) was performed using the yeast UniProt database [20,21]. The FAIMSpro interface was utilized with a dispersion voltage of 5000 V and compensation voltages of either −40 V, −60 V, −80 V or −30 V, −50 V, −70 V, with a TopSpeed cycle time of 1 s per CV [22].

### 2.7. Mass Spectrometry Data Analysis

Spectra were converted to mzXML using MSconvert. Database searches were conducted against *S. cerevisiae* UniProt entries (including reversed sequences for decoy analysis) with a 50 ppm precursor ion tolerance and 0.9 Da product ion tolerance to enhance sensitivity using Comet and linear discriminant analysis (LDA). Static modifications included TMTpro tags on lysine residues and peptide N-termini (+304.207 Da) and carbamidomethylation of cysteines (+57.021 Da), with methionine oxidation (+15.995 Da) as a variable modification. Peptide-spectrum matches (PSMs) were filtered to a 1% false discovery rate (FDR) using LDA, and proteins were further filtered to achieve a 1% protein-level FDR. Protein quantification summed TMT reporter ion intensities from matching PSMs, excluding those with signal-to-noise (S/N) < 100 or isolation purity < 50%. Reporter ion intensities were adjusted for TMT reagent isotopic impurities as per manufacturer specifications. Protein abundances were normalized to equalize total signal across channels, correcting for loading differences. Relative abundance (RA) was expressed as a percentage, with the summed S/N for each protein across all channels totaling 100. Significant protein abundance changes were identified with a |log_2_ ratio| > 1 and *p*-value < 0.01. Data files have been submitted to the ProteomeXchange Consortium via the PRIDE repository [23].

## 3. Results and Discussion

### 3.1. Sample Preparation Was Performed in Parallel with Two Lysis Methods

We evaluated the effectiveness of two protein extraction methods, detergent-based (Y-PER) and mechanical (beat beating) lysis, for profiling protein abundance across wild-type and single gene-deleted yeast strains (specifically, *siz1*Δ and *nfi1*Δ). Using isobaric labeling and LC-FAIMS-MS3, we compared the protein profiles to reveal insights into cellular processes and functional changes associated with these deletion strains.

**Figure 1:**
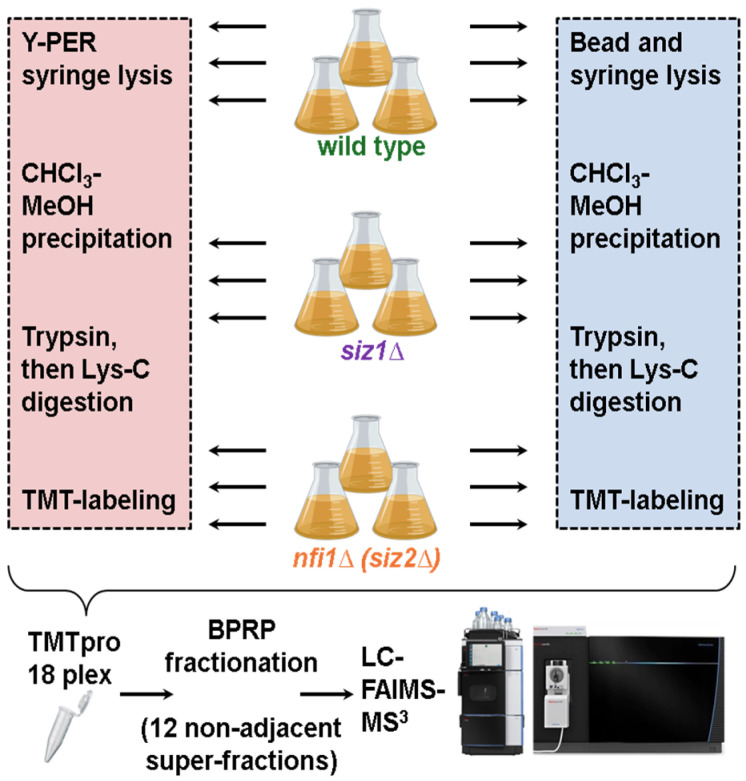
Workflow comparison for protein extraction and analysis. Yeast samples, including wild-type, *siz1*Δ, and *nfi1*Δ (*siz2*Δ) strains, were subjected to two protein extraction methods: Y-PER detergent-based lysis and mechanical bead-based lysis. Both methods included CHCl_3_-MeOH precipitation, trypsin and Lys-C digestion, and TMT-labeling. Further sample processing consisted of pooling the TMtpro-labeled samples, BPRP fractionation (12 non-adjacent super-fractions), and LC-FAIMS-MS/MS to investigate global protein abundance differences.

We outlined the experimental workflow in Figure 1. Yeast cultures of wild-type and the *siz1*Δ and *nfi1*Δ deletion strains were harvested in exponential growth phase (OD600~0.8) and divided for lysis with Y-PER reagent or bead-based lysis. For both methods, lysis was followed by chloroform–methanol precipitation to effectively isolate proteins. Samples then underwent double enzymatic digestion first with Lys-C and then trypsin, after which isobaric labeling with TMTpro-reagents was performed [24]. The pooled sample (for both extraction strategies) was subjected to BPRP fractionation into 96 fractions that were concatenated into 24 superfractions [25] to reduce sample complexity. Data were then acquired using LC-FAIMS-MS3 on an Orbitrap Eclipse mass spectrometer to profile proteomic changes associated with each strain and discover any impact of the extraction method.

### 3.2. Hierarchical Clustering and Principal Component Analysis Revealed Distinct Impacts of Lysis Methods on Protein Profiles

**Figure 2:**
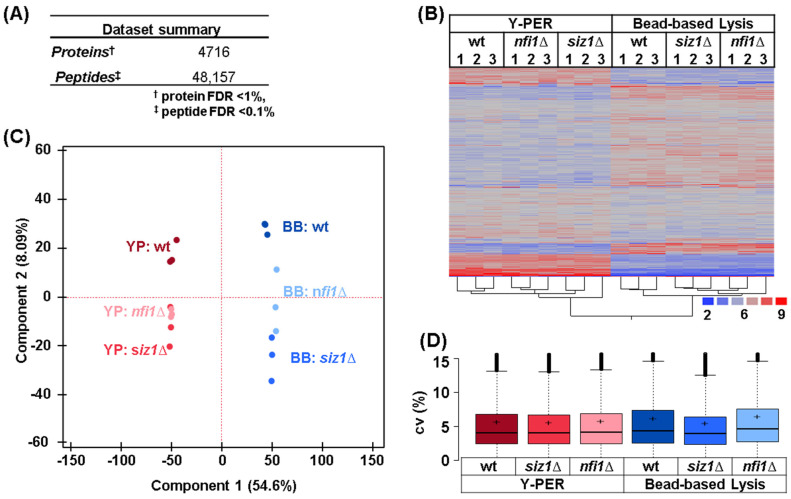
Comparative analysis of protein extraction methods. (**A**) Dataset summary of quantified proteins and peptides. (**B**) Heatmap showing hierarchical clustering of protein abundance across biological replicates (1, 2, and 3) under both extraction methods. (**C**) Principal component analysis (PCA) depicting the variance among wild-type (wt), *nfi1*Δ, and *siz1*Δ strains using Y-PER (YP) and bead-based lysis (BB) methods. (**D**) Box plot summarizing the coefficient of variation (cv) for each strain and method, indicating measurement precision.

We profiled 4716 proteins (Table S1) that were assembled from 48,157 peptides (Table S2) of which 34,016 were unique in sequence (Figure 2A). As such, wee profiled the relative abundance of nearly 80% of the yeast proteome. Using the protein abundance measurements, we performed hierarchical clustering analysis (HCA) with Ward’s linkage and the Euclidian distance metric (Figure 2B). Samples clustered first by replicate, as expected, and second by the lysis method. The fact that the clustering was by the lysis method, rather than by strain, indicated either that minimal difference was observed among the proteome of the analyzed strains and/or the lysis method severely affected the proteome abundance measurements.

We also performed principal component analysis (PCA) on this dataset. The PCA plot illustrated clear segregation of samples based on the extraction method and strain (Figure 2C). The data showed that the first principal component (lysis method) was able to explain over 54% of the variance, while the second principal component (strain) could explain only ~8% of the variance. These data were consistent with the HCA results. We also noted the tighter clustering among replicates for the Y-PER-lysed samples compared to those that were mechanically lysed by beads, implying slightly higher variability in the mechanically lysed sample. To investigate further the variability in our dataset, we plotted the distribution of the coefficient of variation (cv) for the triplicates of our six different sample types (Figure 2D). These data showed that across the dataset, the cv remained low (<5%) with no significant difference among the six sample types. Our findings supported further that high precision data were generated regardless of the sample lysis method used.

### 3.3. Detergent-Based Lysis Demonstrated a Slight Enhancement in Protein Extraction Efficiency

**Figure 3:**
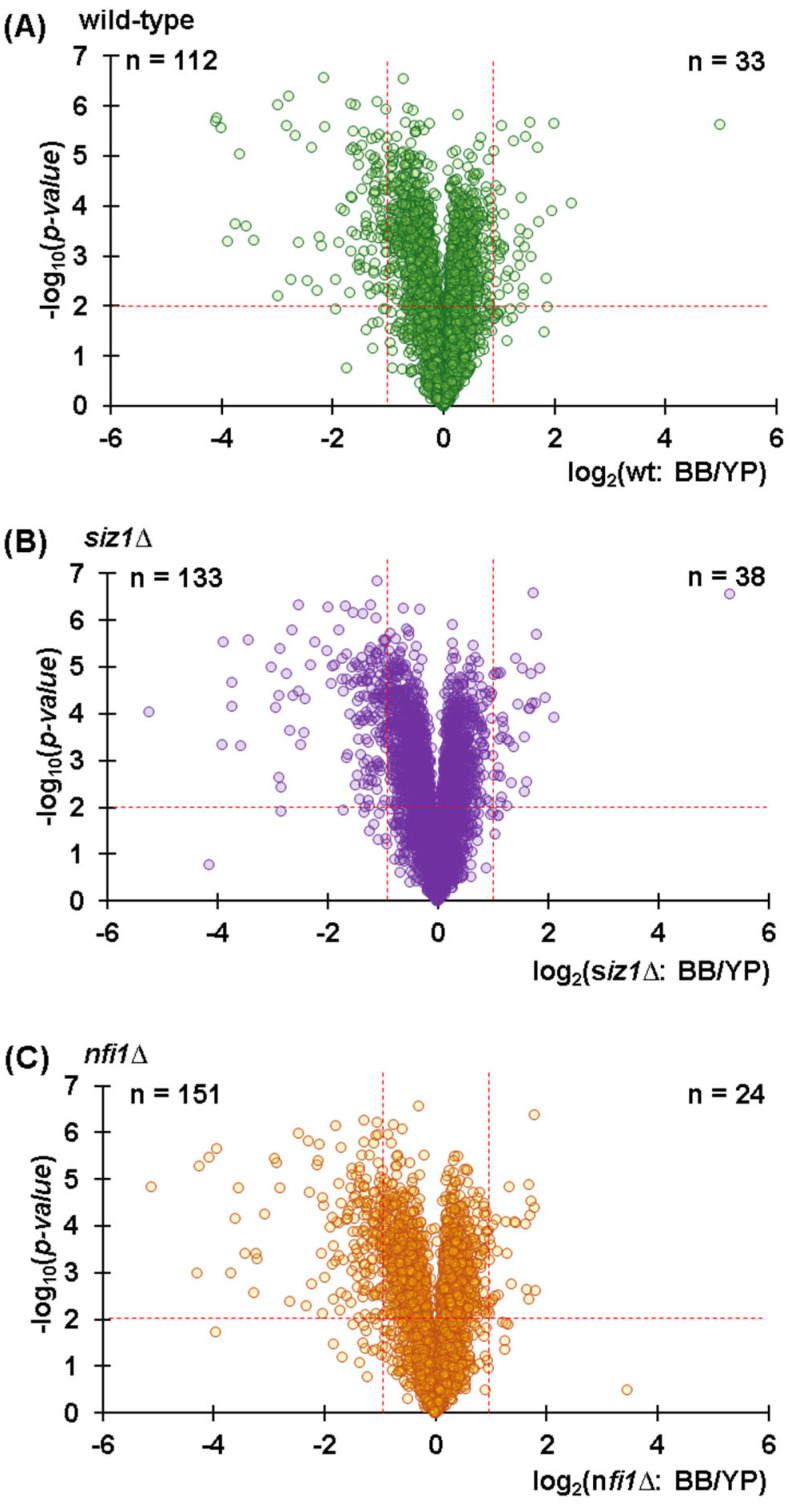
Volcano plots illustrating differential protein abundance between extraction methods. (**A**) Wild-type (wt), (**B**) *siz1*Δ, and (**C**) *nfi1*Δ strains, comparing bead-based lysis (BB) to Y-PER (YP). The plots show log_2_ fold changes versus −log_10_ *p*-values, highlighting significant differentially abundant proteins.

We next compared the two methods to understand which had a higher efficiency in extracting proteins. One benefit of using isobaric tagging was that no missing values were measured within a sample multiplexing experiment; thus, we cannot simply count the difference in the number of proteins quantified when using each method. We must indeed rely on fold changes between the two methods to assess which extracted more protein. We defined a significant change as |log_2_ ratio| > 1 and *p*-value < 0.01. We compared the TMT abundance ratios between samples lysed with the Y-PER reagent and those lysed mechanically by bead-based lysis across all the three yeast strains used in this study. We illustrate the data with volcano plots, where the x-axis represents the log_2_ ratio with respect to wild-type and the y-axis indicates the −log_10_ *p*-values, to highlight the statistical significance of protein abundance changes. The data showed that in the wild-type strain, 112 proteins had lower abundance in mechanically lysed samples, while only 33 proteins were more abundant compared to the detergent-based lysed samples (Figure 3A). Likewise, the deletion strains *siz1*Δ (Figure 3B), and *nfi1*Δ (Figure 3C), showed similar differences as 133 and 155 proteins were of significantly lower abundance, while only 38 and 24 proteins were of significantly higher abundance in the *siz1*Δ and *nfi1*Δ deletion strains, respectively. These data support that both lysis methods performed similarly, with less than 5% of proteins manifesting fold changes ≥2X when we compared these methods. 

### 3.4. Deleting SUMO E3 Ligases SIZ1 and NFI1 Resulted in Minimal Proteomic Alterations, Indicating Potential Redundant Function

**Figure 4:**
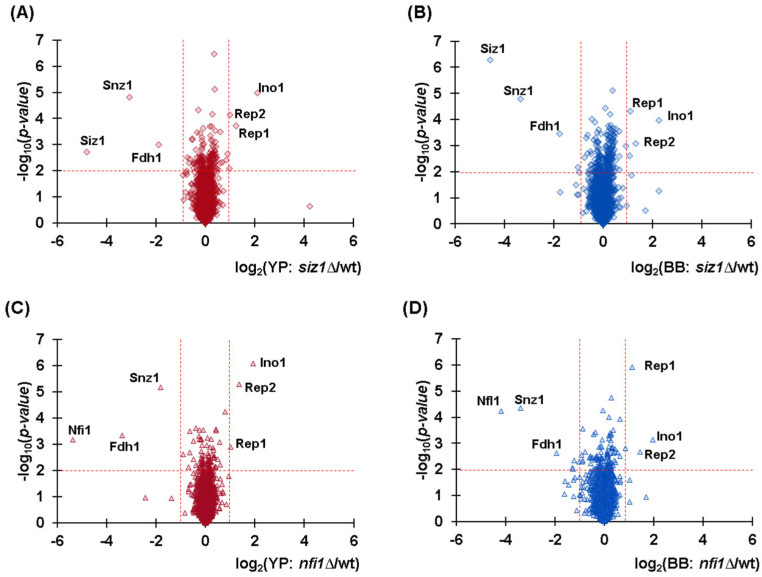
Volcano plots indicating differential protein abundance in knockout strains compared to wild-type (wt). The comparisons with respect to wild-type are (**A**) *siz1*Δ with Y-PER (YP) protein extraction, (**B**) *siz1*Δ with bead-based lysis (BB), (**C**) *nfi1*Δ with Y-PER protein extraction, and (**D**) *nfi1*Δ with bead-based lysis. Labels identify prominent proteins with significant changes in abundance.

In *S. cerevisiae*, three SUMO E3 ligases have been identified: the main SUMO E3 Siz1, its paralog Nfi1 (also known as Siz2), and Mms21. As such, we postulated that deletion of one Sumo E3 would result in significant proteome remodeling when compared to wild-type cells, as SUMOylation has been shown to promote protein degradation. Similar to our comparison between lysis methods, we constructed volcano plots to analyze differential protein abundance in yeast deletion strains compared to the wild-type, using both lysis methods. We first examined the protein abundance differences in the *siz1*Δ strain compared to wild-type for cells lysed with the Y-PER reagent (Figure 4A). Here, we determined three proteins to be statistically higher in abundance, namely, Ino1 (INOsitol requiring), Rep1 (REPlication 1), and Rep2 (REPlication 2). We also noted three proteins with lower abundance, specifically, Snz1 (SNooZe), Fdh1 (Formate DeHydrogenase), and Siz1 itself. Notably, in isobaric tag-based experiments, measuring signal (albeit low signal) is common when a given gene is deleted due to the phenomenon of interference [26]. However, as expected, the most downregulated protein in all comparisons was the one presumably deleted in the strain. We then examined the abundance differences obtained with data from the mechanical lysis method (Figure 4B). As expected, the same proteins were observed to be differentially abundant, although with slight differences in variance and fold change. These data supported both lysis methods as being equally effective in generating reproducible data. We then performed a similar comparison using the *nfi1*Δ strain compared to wild-type. As with *siz1*Δ strain, for both detergent (Figure 4C) and mechanical lysis (Figure 4D), many of the same proteins were dysregulated. Specifically, Ino1, Rep1, and Rep2 were significantly higher in abundance, while Snz1, Fdh1, and this time Nfi1 (which was the deleted protein) were significantly lower. These data again supported the equivalence of the lysis methods. Considering that the same proteins were dysregulated in both the *siz1*Δ and *nfi1*Δ strains, each paralog appears to compensate for the SUMO E3 ligase activity in the corresponding deletion strain, suggesting that these two enzymes likely work redundantly.

Deletion of SUMO E3 ligases *SIZ1* and *NFI1* in *S. cerevisiae* led to minimal proteomic changes, which may reflect the limited number of proteins sumoylated by these ligases in exponentially growing cells or their functional redundancy. However, it is possible that more substantial proteomic changes may be observed under stress conditions, as previous studies suggested that protein sumoylation increases in response to cellular stress [27]. Moreover, *S. cerevisiae* has a relatively small pool of sumoylated proteins, particularly those targeted by Siz1p and Nfi1p [27]. Sumoylation also often regulates protein localization or signal transduction rather than degradation, which could explain the observed subtle proteomic shifts [28]. These considerations suggest that the minimal alterations in *siz1*Δ and *nfi1*Δ strains, as observed here, may stem from specific roles of sumoylation rather than broad proteomic restructuring.

### 3.5. Several Proteins Show Significant Abundance Differences Across Strains

**Figure 5:**
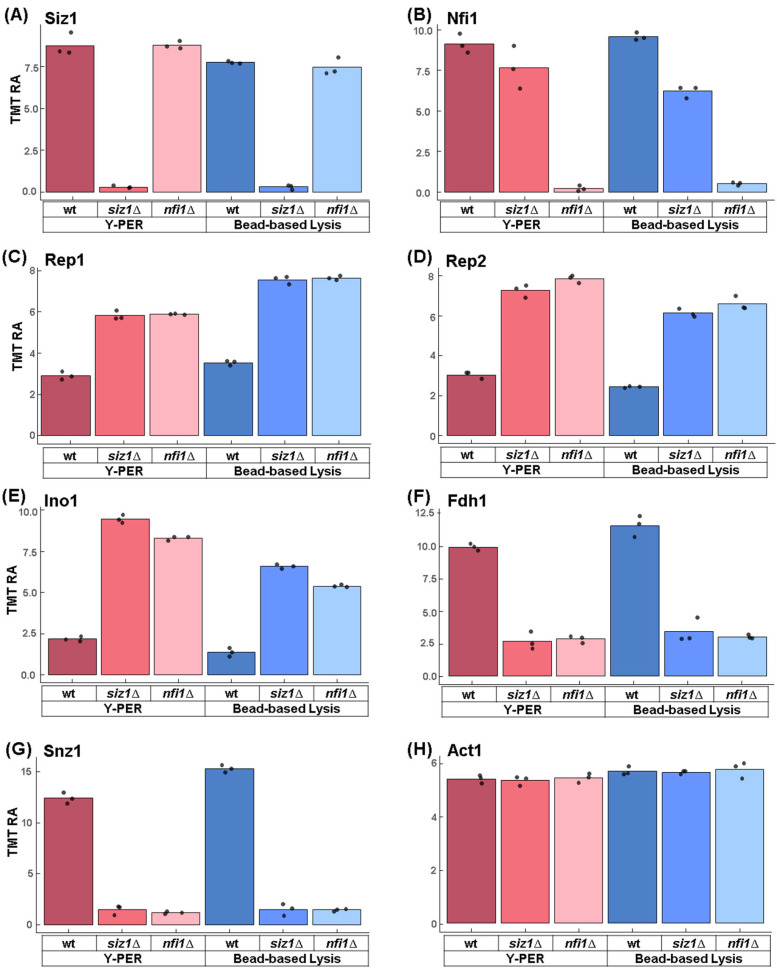
Targeted analysis of selected proteins among extraction methods and strains. Bar plots depict TMT relative abundance (TMT RA) for (**A**) Siz1 (SAP and mIZ-finger domain 1), (**B**) Nfi1 (Neck Filament Interacting 1), (**C**) Rep1 (REPlication 1), (**D**) Rep2 (REPlication 2), (**E**) Ino1 (INOsitol requiring), (**F**) Fdh1 (Formate DeHydrogenase), (**G**) Snz1 (SNooZe), and (**H**) Act1 (Actin). The comparisons are made across wild-type, *siz1*Δ, and *nfi1*Δ strains for Y-PER and bead-based extractions.

We first verified the deletion strains across all samples. The Siz1 protein was, as expected, absent in the *siz1*Δ strain, thereby confirming the deletion (Figure 5A). The wild-type strain showed similar Siz1 abundance across both extraction methods. Interestingly, Siz1 maintained baseline levels and was not upregulated as it may have been expected to compensate for the absence of the Nfi1 protein in the *nfi1*Δ strain. Next, we examined Nfi1, which, again as expected, was nearly absent in the *nfi1*Δ strain (Figure 5B). The wild-type strain exhibited consistent Nfi1 levels, regardless of extraction method. Unexpectedly, we observed a decrease in the abundance of Nfi1 in the *siz1*Δ strain, which could suggest that Nfi1 could work more efficiently when Siz1 was absent to compensate for the absence of Siz1. These data support that Nfi1 and Siz1 are related in function and pathway, and the efficacy or regulation of Nfi1 was altered due to the absence of Siz1.

Next, as we observed minimal proteomic profile alterations, we highlight the function of five proteins showing a significant difference in abundance for both deletion strains. Three of these proteins demonstrated an increase in abundance in deletion strains compared to wild-type yeast, suggesting that they could be targeted by SUMOylation for degradation. Rep1 and Rep2 are important to amplify the two micron plasmids and are known to be SUMOylated [29]. Rep1’s abundance profile was nearly identical in the two deletion strains, with both strains showing an increase with respect to wild-type with slight variation between lysis methods (Figure 5C). Like Rep1, Rep2 maintained consistent abundance levels in the deletion strains, which were higher compared to the wild-type. As in Rep1, the Rep2 profile showed a slight difference in abundance between the lysis methods, but demonstrated an inverse trend (Figure 5D).

Similarity, Ino1, which is essential for inositol biosynthesis and lipid metabolism [30], also exhibited higher abundance relative to the wild-type strain in both deletion strains. Here, however, a slightly greater increase in Ino1 abundance was measured in the *siz1*Δ strain compared to the *nfi1*Δ strain, in similar proportion across both lysis methods. This finding may reflect a paralog-related difference in upstream regulation of pathways related to lipid metabolism and/or membrane synthesis (Figure 5E). In addition, the Y-PER reagent showed ~25% higher isolation of this protein compared to mechanical lysis.

Moreover, two proteins showed a decrease in abundance in the deletion strains compared to wild-type, specifically Fdh1 and Snz1. Fdh1 is involved in metabolic processes like alcohol and aldehyde detoxification [31]. The relative protein abundance of Fdh1 only deviated slightly between extraction methods and deletion strains, reflecting potential differences due to the two gene deletions (Figure 5F). Snz1, which is involved in vitamin B6 biosynthesis and stress response [32], showed a similar protein abundance profile as Fdh1, but with the deletion strains now displaying a greater decrease in abundance compared to wild-type (Figure 5G). Mechanical lysis resulted in a higher proportion for both proteins compared to the Y-PER reagent. Finally, we highlighted Act1, which is a well-conserved cytoskeletal protein involved in cell shape and motility as a control [33]. The data showed that actin (Act1) maintained stable abundance across all strains and methods. This stability reinforced the conclusion that observed differences in other proteins were likely due directly to the specific genetic deletions or the lysis method used, rather than experimental variability.

### 3.6. Functional Enrichment Analysis Underscored the Effects of Gene Deletions on Cellular Processes

**Figure 6:**
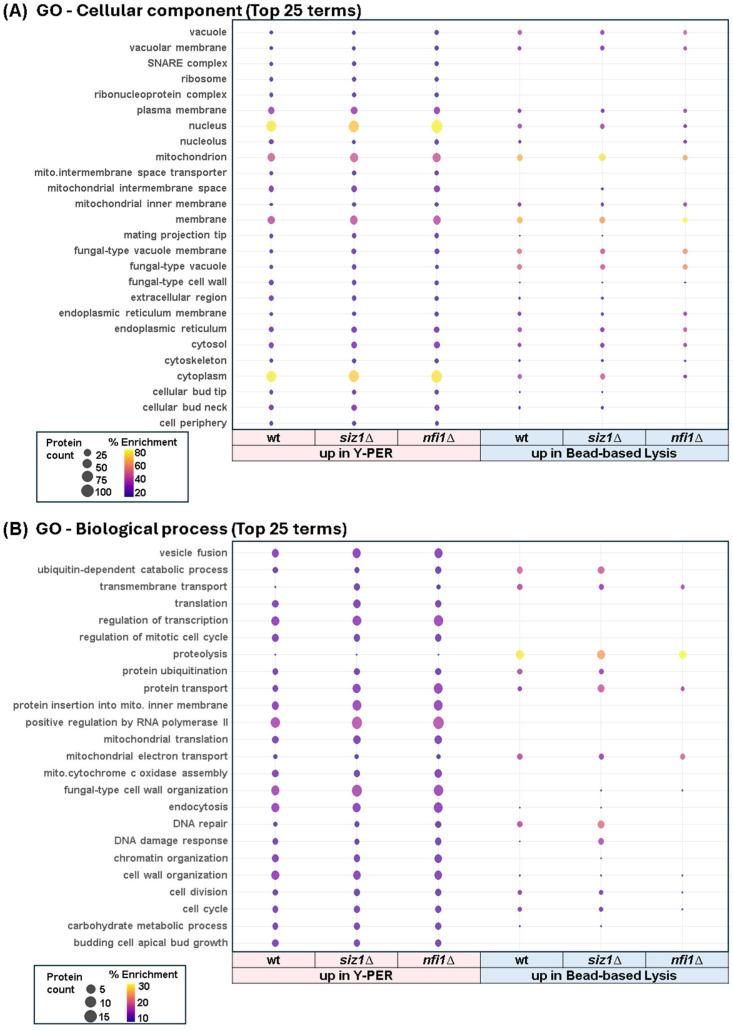
Gene Ontology (GO) enrichment analysis of differentially abundant proteins. (**A**) Top 25 cellular component terms and (**B**) top 25 biological process terms enriched in proteins upregulated in Y-PER and bead-based lysis extracted samples in wild-type (wt), *siz1*Δ, and *nfi1*Δ strains. Dot size represents protein count, and color indicates percent enrichment.

As very few proteins demonstrated significant differences across strains, we explored further the functional and localization differences resulting from the lysis method used. First, we illustrated the top 25 cellular components enriched in proteins with higher abundance when using the detergent-based and mechanical lysis methods across wild-type, *siz1*Δ, and *nfi1*Δ strains (Figure 6A). Remarkably, we observed significant enrichment in mitochondrial and ribosomal proteins in the *siz1*Δ and *nfi1*Δ strains, suggesting robust changes in cellular energetics and protein synthesis in these two strains. Method-dependent variations highlighted how extraction techniques can differentially reveal cellular structures. The detergent-based lysis method uncovered more proteins with distinct GO terms compared to mechanical lysis. In fact, we observed enrichment of membrane structure such as plasma membrane, organelle membranes (e.g., mitochondria inner membrane and endoplasmic reticulum membrane), as well as proteins localized to the bud tip and bud neck (Figure 6A). The Y-PER buffer, which is rich in detergent, likely favored the solubilization and the enrichment of membrane proteins compared to the mechanical lysis.

We also present the top 25 biological processes enriched under the same conditions (Figure 6B). Processes like translation, transcription regulation, and stress response were prominently featured. Both deletion strains showed increased enrichment in transcriptional and translational activities, possibly as compensatory mechanisms for gene deletions. The detergent-based lysis method again uncovered more distinct GO terms compared to mechanical lysis, indicating its efficacy in capturing proteins involved in complex biological processes and stress responses. Together, these panels underscore the impact of gene deletions on cellular functions and the critical influence of extraction methods on protein abundance measurements.

### 3.7. Conclusions and Limitations

Here, we highlighted the impact of protein extraction methods on the analysis of the whole yeast proteome, emphasizing the importance of selecting an appropriate method to efficiently achieve accurate results. Both detergent-based and mechanical lyses were effective in extracting yeast proteins, but the Y-PER reagent required no specialized equipment and resulted in a more streamlined sample preparation workflow. Furthermore, our enrichment analysis revealed that Y-PER can facilitate the extraction of membrane proteins and it can be particularly useful for researchers studying membranes or membrane-related processes such as cytokinesis or membrane trafficking.

We also noted that the analysis of the *siz1*Δ and *nfi1*Δ deletion strains revealed minimal overall proteomic alterations, yet variations in specific proteins, such as Ino1 and Snz1, were present in both deletion strains, suggesting compensatory mechanisms linked to the absence of one of these SUMO E3 ligase paralogs. Proteins such as Ino1, whose abundance increases in the absence of SUMO E3 ligase, represent candidate substrates of these enzymes and they can be further exploited to fully characterize the mechanism of action of these enzymes. 

We acknowledge that combining non-ionic detergents with bead beating may enhance *S. cerevisiae* cell disruption and protein extraction efficiency. Such an approach would use the mechanical disruption of bead beating (if available) to break down yeast cell walls, complemented by the solubilizing capabilities of the detergent to improve protein yield, particularly for membrane-bound proteins. Here, we focused on comparing standalone bead beating and detergent-based lysis methods, and thus did not directly assess this combined strategy. We suggest that future studies may investigate the potential of this hybrid approach.

A limitation of our study is that we relied on consensus protein sequences to build our dataset, which lack proteoforms and their potential functional diversity. Additionally, we did not incorporate the analysis of post-translational modifications (PTMs) such as phosphorylation, ubiquitylation, or acetylation, which are regulatory events influencing protein stability. The detection of these modifications requires enrichment strategies, due to their low abundance. By omitting proteoforms and PTMs, we may have missed important strain-specific differences.

Nevertheless, our findings deepen the understanding of the complex regulatory networks in yeast that are often conserved in higher eukaryotes. In particular, we focused on the impact of SUMOylation in controlling proteome stability, an aspect that has not been explored fully. Furthermore, we provide a framework for future studies on cellular responses to genetic modifications.

## Data Availability

Data files have been submitted to the ProteomeXchange Consortium via the PRIDE repository [20] under dataset identifier PXD063969.

## Supplementary Materials

The following supporting information can be downloaded at: https://www.mdpi.com/article/10.3390/proteomes13030028/s1, Figure S1. Gene Ontology (GO) enrichment analysis of differentially abundant proteins. Table S1. Protein quantification data from the cell lines. Table S2. Peptides quantification data from the cell lines.
