# Supplementary material for: Evaluating Protein Extraction Techniques for Elucidating Proteomic Changes in Yeast Deletion Strains
Source: Proteomes. 2025 Jul 1;13(3):28. doi: 10.3390/proteomes13030028 (PMC12286038; doi:10.3390/proteomes13030028)
Supplement: Supplementary file 1 [file proteomes-13-00028-s001.zip › proteomes-3710244-supplementary Figures.pdf]

Figure S1:

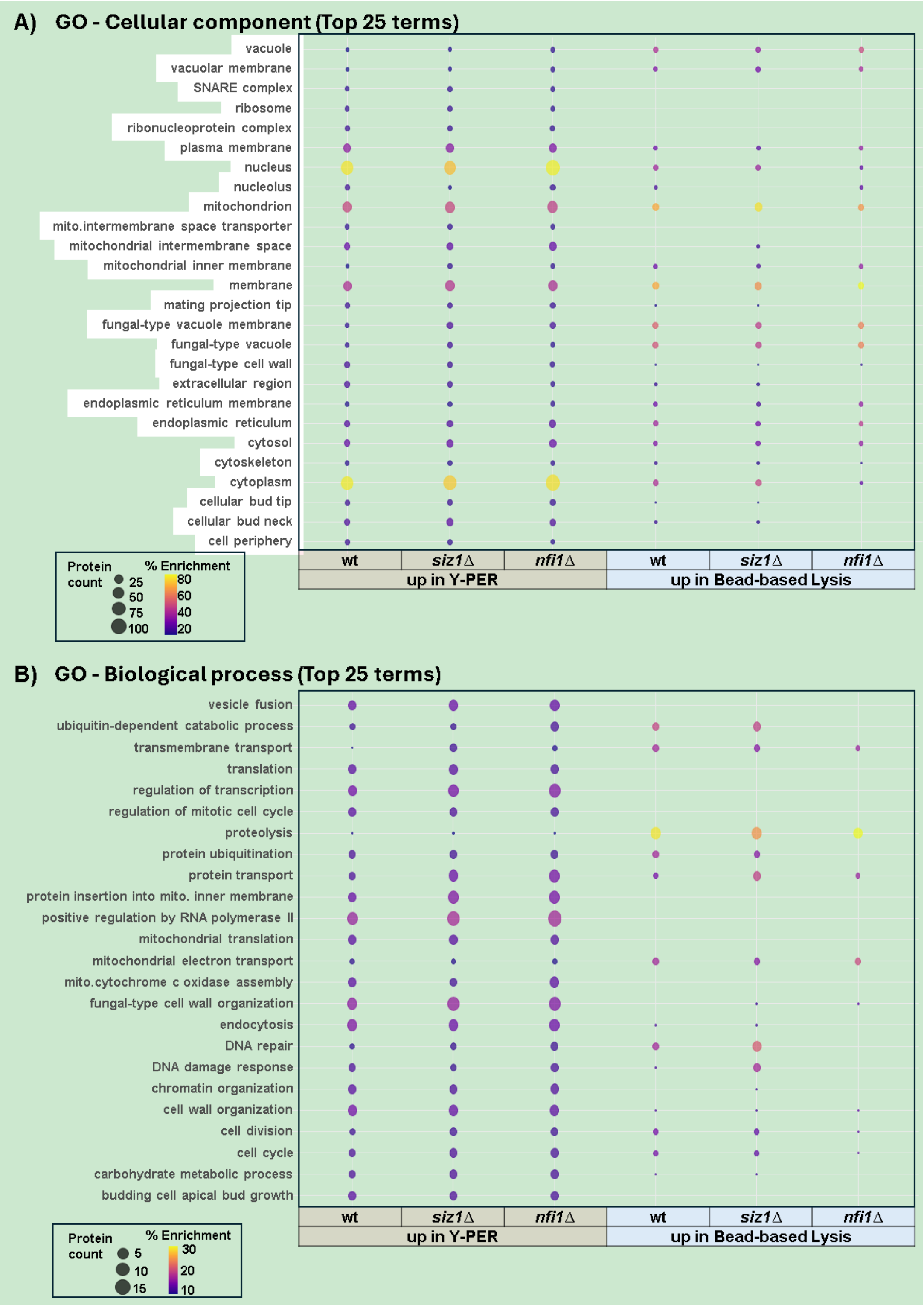

Figure S1: Gene Ontology (GO) enrichment analysis of differentially abundant proteins. A) Top 25 cellular component terms and **B)** top 25 biological process terms enriched in proteins upregulated in Y-PER and bead-based lysis extracted samples in wild-type (wt), *siz1Δ*, and *nfi1Δ* strains. Dot size represents protein count, and color indicates percent enrichment.
